# Continuous Effects of Green Transformational Leadership and Green Employee Creativity: A Moderating and Mediating Prospective

**DOI:** 10.3389/fpsyg.2022.840019

**Published:** 2022-05-06

**Authors:** Mangenda Tshiaba Sidney, Nianxin Wang, Mehrab Nazir, Marcos Ferasso, Abeera Saeed

**Affiliations:** ^1^School of Economics and Management, Jiangsu University of Science and Technology, Zhenjiang, China; ^2^Economics and Business Sciences Department, Universidade Autónoma de Lisboa, Lisbon, Portugal; ^3^Shaheed Zulfiqar Ali Bhutto Institute of Science and Technology, Karachi, Pakistan

**Keywords:** green human resources management, green process engagement, green creativity, green transformational leadership, green innovation strategy

## Abstract

Responding to environmental concerns is a new indication of innovativeness, allowing businesses to achieve competitive advantages by executing innovative activities that benefit individuals and the entire community. Much intention has been retained in this perspective on “green employee creativity.” However, few studies have examined the combined effect of green creativity from relations between people and organizational practices. As a result, we sought to explain the variation in employees’ green creativity by investigating relations of four factors outside of the organizational context (transformational leadership, green innovation strategy, green human resources management—GHRM, and green process engagement) with individual factors (such as employees’ green creativity). Data were retrieved from 150 employees pertaining to electronic companies. Data were statistically analyzed by SmartPLS software. Main results revealed that green transformational leadership positively affects employee green creativity, GHRM, and green process engagement play a significant mediating role in the relation between green transformational leadership and employee green creativity. Furthermore, the green innovation strategy significantly moderates transformational leadership and green process engagement. This effect is improved when the level of green innovation strategy is high rather than low.

## Introduction

With the growth and prosperity of the manufacturing industries in Kinshasa, Congo (Pasha et al., 2020), the government is becoming progressively concerned about environmental issues (Marijnen, 2017). Governments and businesses are trying to explore the path of sustainable growth in response to environmental issues.

While green business models were primarily focused on capital efficiency and maximizing the efficiency of environmental performance, newer models place more emphasis on growth optimization, production cycle, and post-design application, resulting in greater sustainability (Enongene and Fobissie, 2016). A previous study found that companies need to do more than integrate environmental strategies into their product development processes while also trying to benefit from these strategies in the form of increased revenue and improved wellbeing for both parties (Chen, 2011). As opposed to ordinary innovation (Luo and Zhang, 2021), green creativity focuses on the environmental friendliness and long-term viability of products, services, and behaviors (Chen and Chang, 2013). Through the effectiveness of green creativity, businesses and society can attain environmental sustainability (Mittal and Dhar, 2016). Furthermore, green innovation is a key for dealing with global ecological sustainable changes, and it may also help companies achieve a competitive edge (Chen et al., 2012).

Creativity is the ability to come up with original, novel, and valuable ideas (Wyer et al., 2010). An essential factor in business advancement is promoting innovation, and creativity enables companies to stay relevant and adaptable in a dynamic and constantly changing economic climate (Bos-Nehles et al., 2017). Although global warming has recently influenced global issues, like pollution, it cannot be overlooked its effects on economic development. In adapting to ecological challenges, business innovations can be deliberated as a new expression of improvement to give organizations an advantage over their competitors by engaging in inventive projects for the greater good (Aboelmaged and Hashem, 2019). All of this takes place against the backdrop of a recent change in the definition of “green creativity.” Green creative work refers to developing innovative and valuable ideas with ecologically conscious inputs to create products, services, processes, and practices for companies (Zhang J. et al., 2020).

The collaboration required to enhance green creativity depends on the different approaches to managing environmental strategies and the corresponding human resource (HR) practices (Al-Hawari et al., 2021). As stated previously, if a company aspires to be more environmentally friendly while simultaneously empowering staff to participate in activities like training, it must create a structure that gives workers, such as trainees and opportunities (Bos-Nehles et al., 2017). The study of green creativity and green human resource management (HRM) in organizations has previously remained stagnant due to a lack of green human resource management (GHRM) research (Al-Hawari et al., 2021). Companies should focus on using environmental strategies with clear and explicit connections to human resources practices that promote and support environmental practices among their employees (Agarwal, 2014). This includes implementing policies to control environmental and sustainable workforce practices.

The previous research focuses primarily on the organizational drivers of green creativity (Tierney and Farmer, 2004). To better understand green creativity, we must study it on both a macro- and micro-level. At the macro-level: the emergence of green business practice is at the foundation of an organization. At the micro-level: the individual’s incorporation of green practices is fundamental to a company. Most prior research has been dedicated to examining the internal mechanics of green creativity (Mittal and Dhar, 2016). This means that businesses face external pressures that cause employees and leaders to pursue green creativity (Lin et al., 2013). Companies must practice green development by implementing an integrated strategy incorporating extensive external collaboration and internal resource integration with stakeholders, commonly referred to as “open innovation” (Chesbrough, 2003).

While, organizations used open innovation for the potential ability to help firms attain external information and resources, compensate for internal inadequacies, reduce research and development uncertainty, and improve learning ability (Tseng et al., 2013). It would assist firms in developing or embedding an innovative external network, expanding living space, and integrating internal and external technology to boost their potential to innovate (Yun et al., 2019). Consequently, organizations might create a corresponding green innovation plan (Eiadat et al., 2008), which illustrates the stimulus of external influences on the organization as the internal action requirements for green innovation. According to studies, a green innovation approach has a considerable impact on long-term creativity (Song and Yu, 2018).

The essential purpose of this study is to recognize the environmental usage of transformational green leadership and green creativity by addressing the intermediary routes and boundary conditions that are found between them. This study consists of three observations: first, the researcher explained the direct relation between green transformational leadership and green creativity; second, we have found that it plays a substantial influence in illustrating that green human resource management contributes to green transformational leadership and green innovation. Finally, we presented a new variable, “green innovation strategy,” classified as a moderator effect between green transformational leadership and green human resource management.

This research develops and tests a model to find the links between the benefits of green transformational leadership and green employee creativity and the mediating influence of GHRM. It will also investigate the role of green innovation strategy in moderating the association between green transformational leadership and GHRM. Although green transformation leadership is critical for increasing employee creativity, it is still necessary to do research that drives this requirement, and without it, green transformation leadership is not attainable (Zhang W. et al., 2020). This is why the world’s largest firms will pay close attention to it. This research investigates the effects of green transformational leadership and green employee creativity on employee retention. The present study will examine the relationship between green human resource management and green innovation strategy and fill the remaining gap by evaluating green human resource management’s direct and indirect impacts.

Table 1 shows the definitions of the main variables to help clarify the hypothesized model. This study demonstrates the comprehensive approach to examining green creativity components, the research model anticipated, and the multiple variables used, which are expected to add to the literature on green creativity.

**Table 1:** Shows the definitions of the main variables to help clarify the hypothesized model.

Terms	Definition
Green Transformational Leadership	Leadership behaviors inspire and motivate members to help them realize environmental goals and leadership behaviors that go above and beyond expectations regarding environmental performance (Chen, 2011).
GHRM	A new approach to the realization of overall HR process, the key features of which are to incorporate ecological objectives into all areas of HRM, beginning with employment planning, continuing with the recruitment of staff, assortment, employee training, employee motivation and development, and finally culminating in their assessment and effect on working conditions (Bombiak and Marciniuk-Kluska, 2018).
Green Human Resource Management	The GHRM refers to HRM practices that promote companies’ ecological and environmental effects and are directly connected to strong environmental strategies and green employee behaviors (Singh et al., 2020).
Green Innovation Strategy	It means to achieve these several aims, which include environmental pollution reduction, resource conservation, waste reduction, and overall environment enhancement, a company may (and should) follow a sustainable approach that takes into consideration external environmental conditions (Ge et al., 2018).
Green employee Creativity	Green creativity is defined as “the generation of new ideas about green products, green services, green processes, or green practices that are deemed original, novel, and useful” (Chen and Chang, 2013). Leadership understands employees’ needs, abilities, and motivations and guides them to contribute green ideas for the future. GHRM motivates its subordinates to think creatively, examine problems from various perspectives, and develop innovative environmental solutions (Chen and Chang, 2013). The term “GCRT” refers to the behavior of employees in the tourism and hospitality industries. They may exhibit a propensity to propose novel methods for achieving environmental goals, offer and encourage green-oriented ideas to improve their firm’s environmental performance, rethink new green ideas, and seek creative solutions to environmental problems. The productive innovation and valuable notions with ecologically friendly effects on manufacturing processes, corporate products, services, and corporate practices will benefit the organization (Jia et al., 2018).

This study demonstrates the comprehensive approach to examine the components of green creativity, the research model anticipated, and the multiple variables used, which are expected to add to the literature on green creativity.

This paper is structured as follows. After this introduction, a literature review on GHRM, transformational leadership, and green creativity are presented. The research methodology and measures are presented in "Results", following the presentation and discussions of the results. The paper ends with a conclusion and cited references.

## Underpinning Theory

### Ability–Motivation–Opportunity Theory

The AMO theory is an essential theoretical paradigm in strategic human resource management (Boxall et al., 2007; Gerhart and Fang, 2015). Its defining characteristic emphasizes the influence of human resource systems on overall employee effectiveness and attitudes at the managerial level. According to a general concept, “performance = employees’ ability, motivation, and opportunity to participate,” argued that an HR system that provides appropriate opportunities and platforms for trained and skilled employees is best suited to satisfy organizational interests (Boxall et al., 2007). AMO theory highlights the significance of employees’ abilities, opportunities, and motivations in contributing to managerial performance; this is an assimilating perception that demonstrates how and why the strategy of top management and HRM practices endorse firm performance (Colbert, 2004). Original, novel and valuable ideas created by individuals in an organizational context are frequently referred to as workplace creativity. Workplace creativity can enhance the effectiveness of company performance, or, more precisely, employee creativity is part of the first critical stage of innovation that leads to growth (Shalley et al., 2004).

Skilled and empowered individuals may not always ensure good Sustainability behavioral performance—the importance of giving adequate chances and platforms cannot be underestimated (Boselie et al., 2005). Green creativity, as an essential component of workplace creativity, may be considered as the same activity. As stated previously, if a company aspires to be more environmentally friendly while simultaneously empowering staff to participate in activities like training, it must create a structure that gives workers, such as trainees and opportunities (Bos-Nehles et al., 2017). AMO modifies the attachment of green creativity with ecology measurements in the work environment. The primary explanation is that AMO components will always affect the production of unique and valuable environmental ideas created by combining individual and situational elements (Jia et al., 2018).

Consequently, our study examines workers’ green creativity in the workplace using AMO theory. Because of its tight relationship with the subject of strategic HRM, enhancing GHRM is nearly often framed in terms of a strategic HRM behavioral approach (Schuler and Jackson, 1987). However, leaders’ encouragement and employee participation must be part of the process (Agarwal, 2014). The stakeholders are the people who are the primary concern regarding the execution of different GHRM initiatives since they are in the direct line of business with the processes that influence the company’s overall productivity and competitiveness. Considering this, you must recognize that employee development of green creativity and the execution of GHRM policies are both influenced by leadership (Kim et al., 2017).

### Green Transformational Leadership

Green transformational leadership can significantly help improve employees’ green creativity by promoting individuals and encouraging a green creativity environment (Elrehail et al., 2018). While researchers are still discussing which kind of leadership is helpful in an organization, research has already highlighted what transformative leadership is important at this time (Lin and Hsiao, 2014). Earlier research has shown that transformative leadership components have international universality (Elrehail et al., 2018) and play an essential role in enhancing employee creativity (Li et al., 2014).

Green transformational leadership comprises the qualities of Intellectual motivation, motivation, charisma, and individual consideration (Hameed et al., 2021). Intellectual stimulation can help followers’ cognitive abilities, and make it easier for them to generate problems, conduct research, and think of solutions, making followers more creative (Mansoor et al., 2021). Transformational leadership focuses on increasing employees’ awareness of progressive ideals, like independence, fairness, honesty, and humanism, while encouraging subordinates to put the organization’s needs above their own needs (Aboramadan and Karatepe, 2021). Transformational leaders have four distinct behavioral components: inspirational motivation, charismatic personality, individual attention, and cognitive stimulation, influencing their opportunity to empower followers (Elrehail et al., 2018). When a leader inspires with a desirable vision and high expectations, individuals will become more motivated to help realize the goals and visions of the organization. This, in turn, helps people be more committed to the firm’s mission and goals and makes them more willing to provide ideas and suggestions (Mansoor et al., 2021).

Green transformation leaders attend to individual subordinates’ specific needs, guide and support them, and infuse them with a sense of belonging (Mittal and Dhar, 2015). Transformational leaders can inspire their followers by spreading ideas that respect and trust. Transformative leaders help employees develop innovative ideas by encouraging them to express their thoughts and interest in new ideas. According to previous research (Gong et al., 2009), transformational leadership strongly impacted employee creativity. As they have massive effects on environmental performance (Andriopoulos, 2001), organizational creativity depends heavily on the leaders and their feasibility (Halbesleben et al., 2003). One of the managers’ main interests is to promote the green creativity of employees to generate innovation (Renwick et al., 2013). In the environmentally friendly structure, “green transformation management” refers to “the process of innovation, which is deemed unique, original and beneficial, on Environmentally friendly products, Eco-friendly Services or Sustainable Processes” (Thompson and Choi, 2006; Renwick et al., 2013). Previous studies have shown that green leadership transformation and green creativity have a positive relationship (Jia et al., 2018).

The first hypothesis raised is presented below:

*H1*: Green transformational leadership is positively related to green employee creativity.

### Green Transformational Leadership, GHRM, and Employees’ Green Creativity

A firm’s green creativity can be significantly affected by implementing sustainable managerial development. Environmentally friendly policy and the use of raw materials more efficiently and even inspire ideas that help the environment, even though they may seem unlikely at first (Eiadat et al., 2008). Transformational leadership profoundly impacts how a business’s HR practices are carried out (Renwick et al., 2013). Leadership significantly influences HRM concepts, objectives, and policy decisions, but implementing HRM practices has become an effective means for senior managers to implement company strategy and visions. While transformational leadership has an optimistic effect on performance management, talent management, and employee efficiency, research indicates that the intellectually inspired aspect of this leadership style influences each of these three aspects differently (Khattak et al., 2020). This means that a transformational good management team interacts with green objectives to HRM and creates a change if an organization pursues an environmental objective. The first type of ability-enhancing green HR practices focuses on green recruitment and selection. Specifically, promoting environmental awareness and pro-environmental behavior when recruiting is green recruitment and selection. Since candidates with ecological knowledge and consumer skills appear to be oppressed at work, compared to not employed subordinates, candidates evaluated by this strategy are more likely to understand the corporate sustainable organizational objectives that should boost their desire for green creativity. It is used to describe training activities that promote awareness and help employees develop protecting the environment skills, providing an opportunity for employees to learn environmental protection techniques and create an environmentally friendly organizational culture in which everyone is encouraged to be part. GHRM expresses a company’s position on environmental protection and helps executives focus on the process while encouraging employees to perform measures in the workplace that lower environmental pollution (Mishra et al., 2014). Thus, we propose the following hypothesis:

*H2*: Green transformational leadership is positively associated with GHRM.

### Green Transformational Leadership; Create Process Engagement, and Employees’ Green Creativity

Earlier research has demonstrated that transformational leadership can significantly impact an organization’s creativity (Li et al., 2014). However, it is important to study how green transformational leadership affects green creativity, especially from an environmental perspective. Engagement in the creative process has three key elements: generating new ideas (Nave and Franco, 2019; Zhang J. et al., 2020). In the conservation of resources theory, employees should contribute and acquire many resources, thus allowing for creative problem-solving. The resource integration of leaders involves creating an environment that fosters creativity and encourages participation in group projects, leading to increased responsibility and a sense of collective purpose within their subordinates (Reiter-Palmon and Illies, 2004). According to Mumford, transformational leadership offers followers the intelligence to accept new ideas.

Additionally, it assures subordinates to take innovative actions without fearing that those changes will threaten previously established objectives, methods, relationships, and norms. This is in addition to the concept of information processing (Salancik and Pfeffer, 1978), which argues that employees must organize and enhance knowledge acquired by integrating resources to increase their level of creativity (Amabile, 1988; Jaiswal and Dhar, 2015). There are three stages in the creative process: first, establishing the investment strategy; second, to conceive and improve innovative concepts; and third, to combine information to generate alternative solutions. According to their research, “laborious cognitive processing is required to solve problems” creatively (Reiter-Palmon and Illies, 2004). Once employees’ innovative activities are not effectively organized, the quality of the solutions may be influenced (Zhang and Bartol, 2010; Kusurkar et al., 2011).

*H3*: Transformational leadership is positively associated with green process engagement,

### Green Transformational Leadership, Green Process Engagement, GHRM, and Employees’ Green Creativity

Organizational environmental strategy can positively impact a company’s green creativity (Smith, 2004). An environmentally friendly work environment encourages the use of resources efficiently while also accepting even unorthodox ideas that lead to improvements in environmental performance. GHRM reflects a company’s environmental strategy and orientation, encourages executives to pay more attention to the process, and encourages employees to reduce environmental pollution (Yang, 2007; Mishra et al., 2014). We propose that GHRM serves as a bridge between transformational leadership and employees’ green creativity.

Transformational leadership expresses and influences a business’s HR practices (Pereira and Gomes, 2012). Leaders significantly influence the decision-making process when implementing HRM theories, objectives, and policy decisions (Hai et al., 2020). Still, they are essential for top management to formulate business plans and visions (Choudhary et al., 2017). Numerous studies of transformational leadership indicated that growing innovative thinking in leaders has a substantial effect on performance measurement, talent management, and employee productivity (Khattak et al., 2020). Organizations that pursue environmental goals can effectively communicate them to human resources and positively impact them. While numerous studies showed a correlation between HRM and creativity (Laursen and Foss, 2003), few studies demonstrated a link between GHRM and innovation at the discrete level (Bos-Nehles et al., 2017). Using the AMO framework, we found that GHRM practices (e.g., goal setting, comportment, and development) can be classified as GHRM practices that strengthen skills, motivate, and improve opportunities. Green creativity is an essential aspect of innovation, and it involves all employees (Ali Ababneh et al., 2021; Mangenda Tshiaba et al., 2021).

Transformational leadership is all about establishing the company’s HR management practices through the senior managers’ beliefs, attitudes, beliefs, and behaviors (Ali Ababneh et al., 2021). When it comes to the formulation of HRM concepts, goals, and policies, leaders have a strong influence on the decision-making process (Abudaqa et al., 2019). Still, when it comes to implementing practices, they serve as a significant basis for top managers to formulate business strategies and visions (Manafi and Subramaniam, 2015). Green hiring and selection and green training are primary GHRM practices that increase employee ability (Hameed et al., 2021). People who approach their job using environmental awareness and good environmental behavior are likely to be green (Singh et al., 2020). This measure differs from employees who lack ecological awareness and ecological protection skills in two ways: first, employees should be well qualified to recognize the company’s environmental management purposes and thus can apply their creativity toward solving problems pertaining to environmental protection, which engages them and motivates them to act in a green manner; and second, subordinates with this metric in place are more in tune with the business’s overall environmental concerns, resulting in better problem-solving abilities (Rizvi and Garg, 2020). Using the term “green training” can help create an environment where employees learn about environmental protection and increase their sensitivity toward the environment (Jia et al., 2018). This empowers them to learn environmental protection skills and creates an environment where all employees feel comfortable participating.

Many lines of evidence favor our prospect of an optimistic relationship in this study. We hypothesize that transformation leadership mediates the relationship between employees’ green creativity and their use of GRHRMs. Finally, green GHRM practices are defined as opportunity-enhancing (Singh et al., 2020). Workplace engagement includes the promotion of environmental sustainability (Peng et al., 2020). In this regard, there is a venue for employees to discuss environmental management and the capacity for them to participate in decision-making while having some separate identity and freedom in their work tasks, which can help foster international hospitality between organizations and their employees (Moin et al., 2021), encouraging employee participation in environmental citizenship behaviors and inspiring new ideas about environmentalism. Finally, Green involvement means employees have the opportunity to actively manage environmental issues and are given some degree of independence and flexibility in their daily tasks (Peng et al., 2020), which can promote positive workplace relationships and encourage more workplace-based behaviors that support the organization’s environmental goals, while also encouraging employees to create even more environmentally friendly ideas (Li et al., 2020).

Thus, we proposed the hypothesis.

*H4*: GHRM mediates the relationship between green transformational leadership and green creativity.

*H5*: Green process engagement the relationship between green transformational leadership and green creativity.

### Green Innovation Strategy Moderates the Linked Relationship by Creative Process Engagement

Companies with a green innovation strategy inspire waste reduction and develop corresponding rules to monitor the efficient use of raw materials to reduce costs (Eiadat et al., 2008). Manufacturing success and increased urbanization have led to growing environmental challenges (Chan et al., 2005). The government has participated in several distinctive perspectives, such as introducing policies and regulations on environmental protection adjusting environmental taxation, emphasized two issues related to the environmental issues: traditional production, such as steel, has to consider how energy conservation and emission reduction can be carried out and reduced (Ahmeda et al., 2020). Therefore, the way a green innovation strategy is to be implemented has drawn much interest from studies (Su et al., 2020). This strategy showed companies have a more comprehensive range of environmental sustainability capacities by adjusting their manufacturing practices and business initiatives to improve their working performance by implementing environmental management systems (Cai et al., 2020).

However, few researchers explored how green strategies for innovation influence green creativity. The Green Innovation Strategy has strength in earlier studies by linking green transformation leadership and creative process engagement (Li, 2014). The absence of financial and human resources can be the core barrier in the innovation process of employees (Wang et al., 2020). Thus, suitable resources are beneficial to green creativity development (Freeman, 2013). Multi-lateral coordination, including market demand, technological elevation, and policy forecasting, must also be used for green innovation resources. Businesses should thus consider adopting a green innovation strategy to reach particular needs (Wang, 2019). It allows insiders to understand external needs to inspire thinking, improve resource utilization of resources and thus promote green creativity (Song and Yu, 2018). However, this approach is generally articulated by the organization’s management committee, and the team leader can usually affect the green innovation strategy of the company [63]. Earlier research into green innovation strategies emphasizes the organization’s perspective and hardly discusses the role of the green innovation strategy (Ahmeda et al., 2020). This study believes that a green innovation strategy moderates the relation between transformation leadership and creative process engagement to fill this gap.

*H6*: Innovation strategy moderates the relation between the transformation leadership and creative process engagement.

**Figure 1:**
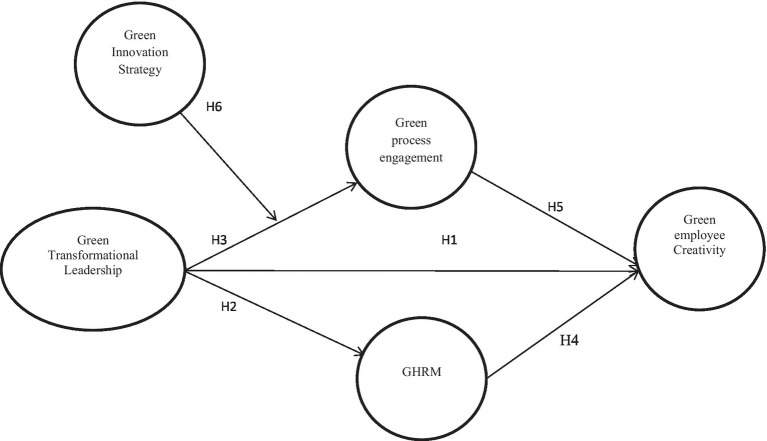
Research framework.

Then, the research framework is presented in Figure 1 as follows.

## Research Methodology

In this study, we have surveyed through a questionnaire to test the hypothesis of electronic firms in Kinshasa, Congo. There are two reasons behind the choice of electronic companies. Firstly, air, soil, or water resources can be polluted by the electronic production process and the product itself, giving stringent environmental protections for such companies. Electronic companies can improve their sustainable development and promote sustainable outcomes that greatly benefit the environmental challenges. Secondly, environmental problems can be aggravated due to the climate factors in Kinshasa, Congo. Therefore, it is essential to study the influences of transformation leadership, innovation strategy, and GHRM to stimulate green creativity in employees when ecological trends present them with a significant challenge. We used a survey to look at transformation leadership, innovation strategy, and GHRM to stimulate green creativity for our employees. Every participant was informed of voluntary participation and confidential response. The questionnaires were subsequently mailed randomly to the company *via* post-payment envelopes.

Collecting primary data is very significant and time-consuming to check the consequence of green transformational leadership on green employee creativity, especially in the Kinshasa province of the democratic republic of Congo, from my own experience. First, we sent the questionnaires to the HR department of the targeted companies (*n* = 170) in the Kinshasa province of the democratic republic of Congo. In the meantime, HR managers circulated a survey questionnaire that requested staff to assess their green creativity. In this study, 170 questionnaires were distributed, and 150 valid answers were received. The number of research samples supported the Slovin formula (Praditya, 2020). The response rate was 80%. The descriptive statistics of the 150 employees are in Table 2. Most participants (64%) were male, aged between 31 and 40 years (46%).

**Table 2:** Demographics.

Variable	Category	Frequency	%
Gender	Male	96	64
	Female	54	36
Age	20–30	25	16.7
	31–40	69	46
	41–50	31	20.7
	51–60	18	12
	61-above	07	4.6
Qualification	Bachelors	31	20.7
	Masters	66	44
	Postgraduates	35	23.3
	Diplomas	10	6.7
	Others	08	5.3

## Measures

Respondent data was collected *via* a structured questionnaire which consisted of different segments relating to the social-economic factors of respondents and relating to the main variables (green transformational leadership, green process engagement, green human resource management, green strategic innovation, and green employee creativity). We added one section to the questionnaire to examine the demographic characters of employees. We also measured the level of green employee creativity by the company’s employees to determine the substantial perspective regarding green human resources and green transformational leadership. Respondents gave their opinion based on a five-point Likert scale from 1 to 5: 1 = strongly disagree; 2 = disagree; 3 = neither agree nor disagree; 4 = agree; 5 = strongly agree.

The usefulness of this research hugely depends on the efficiency of data collection and analysis. The existence of multicollinearity indicated that redundant information is being used in the model (Raykov and Marcoulides, 2006), which may easily escalate to unreliable regression coefficient estimations. SPSS used to find multicollinearity among variables that are connected. A list of measured items and the sources of each part is presented separately with Table 2. Coding operation is then undertaken at this stage, through which the categories of data are transformed into symbols that are tabulated and counted. Collected data coded and modeled using SmartPLS/SPSS Software (Hair et al., 2011). Next, the hypothesized structural relationship among observed variables is assessed to test the hypotheses based on the structural model-direct model, mediation, and moderating model (Aiken et al., 1991). This presented two structural models used for analysis. Bootstrapping is used for the mediation/indirect hypothesis (Preacher and Hayes, 2008). The bootstrapping technique is a supplementary method proposed for mediation analysis to determine the magnitude of the indirect effect, determine the statistical significance of the estimate, and get a sense of the distribution of the estimated parameter (Mallinckrodt et al., 2006).

## Results

SEM is a valuable and widely recognized method of data analysis in social science (Hair et al., 2014, 2017). The current study used the partial least square structural equation modeling with SmartPLS 3 software to evaluate the proposed hypotheses. The PLS-SEM method enables researchers to manage multi-construct models with many concepts, elements, and structural paths without imposing hypotheses on data distribution. A cause and effect SEM predictive method emphasizes the estimate in model evaluation (Ab Hamid et al., 2017; Hair et al., 2017; Sarstedt et al., 2017).

### The Measurement Model

We evaluated the hypothesized model in PLS-SEM in two ways. In the first step, we evaluated the measuring model (Outer Model; MME), while the second was a structural model (Inner Model) evaluation was performed (Henseler et al., 2016; Nitzl et al., 2016). Convergent validity (CV), discriminant validity (DV), and internal consistency (ICR) were determined in MME.

### Convergent Validity and Internal Consistency Reliability

According to the theory of Hair et al. (2017), the CV was evaluated using factor loading. Based on Table 3, in the first model evaluation, the factor loading of all items was higher than the required threshold value of 0.70. Furthermore, according to the results of Table 3, CR and average extracted variance values (AVE) were also higher than the 0.50 criterion value. Consequently, Table 4 showed that the model fulfilled the required CV and ICR benchmarks. The AVE values were higher than 0.50, and CR for all buildings was higher than 0.7 (Hair et al., 2010). According to Fornell and Larcker (1981) criterion, all the constructs fulfilled the DV criteria in Table 5.

**Table 3:** Cross loading.

	GEC	GHRM	GIS	GPE	GTL
GEC1	0.832				
GEC2	0.893				
GEC3	0.887				
GEC4	0.885				
GEC5	0.895				
GEC6	0.871				
GHRM1		0.868			
GHRM2		0.844			
GHRM3		0.870			
GHRM4		0.808			
GHRM5		0.840			
GHRM6		0.868			
GHRM7		0.864			
GIS1			0.910		
GIS2			0.644		
GIS3			0.833		
GIS4			0.940		
GIS5			0.926		
GIS6			0.871		
GPE1				0.895	
GPE2				0.905	
GPE3				0.839	
GPE4				0.929	
GPE5				0.925	
GTL1					0.926
GTL2					0.872
GTL3					0.893
GTL4					0.863
GTL5					0.884
GTL6					0.848

**Table 4:** Convergent validity (CV) and internal consistency reliability (ICR).

	Cronbach’s Alpha	rho_A	CR	AVE
GEC	0.941	0.949	0.953	0.770
GHRM	0.937	0.948	0.949	0.726
GIS	0.930	0.983	0.944	0.740
GPE	0.941	0.948	0.955	0.808
GTL	0.943	0.950	0.954	0.777

**Table 5:** Discriminant validity.

	GEC	GHRM	GIS	GPE	GTL
GEC	0.878				
GHRM	0.305	0.852			
GIS	0.028	0.072	0.860		
GPE	0.432	0.292	0.140	0.899	
GTL	0.305	0.162	−0.005	0.238	0.881

The convergent validity with Cronbach’s alpha, rho_A, the average value extracted (1), composite reliability (2), and confirmatory factor analysis (CFA) was above thrush hold value and acceptable (3). The values for convergent validity should be higher than the thrush hold values; rho_A ≥ 0.7, CR ≥ 0.8, AVE ≥ 0.50, and CA ≥ 0.80 (4). The convergent validity for all variables was acceptable and in the range (5).

The primary method of the Fornell–Larcker criterion was used to measure the discriminant validity and cross-loadings (6). Table 5 shows that the approach of the Fornell–Larcker criterion was fitted to the current research model in discriminant validity.

Tables 6 and 7 show the applied heterotrait–monotrait ratio (HTMT) analysis, which also explored the discriminant validity values (7), and the values were much closer in HTMT path analysis (5). The HTMT value should be less than 1 between factors. To clearly distinguish between the two factors, the HTMT ratio should be less than 1 (5), and the below table shows that all the values were in accordance with the threshold values. Therefore, it is concluded that there was no discriminant validity issue.

**Table 6:** Heterotrait–Monotrait Ratio (HTMT).

	GEC	GHRM	GIS	GPE	GTL
GEC					
GHRM	0.311				
GIS	0.078	0.085			
GPE	0.450	0.303	0.132		
GTL	0.312	0.168	0.043	0.251	

**Table 7:** Direct effects.

	OM	M	SD	*t*-value	*p*-values	Status
GHRM - > GEC	0.175	0.177	0.057	3.096	0.002	Accepted
GIS - > GEC	0.042	0.045	0.018	2.327	0.020	Accepted
GIS - > GPE	0.127	0.133	0.048	2.631	0.009	Accepted
GIS*GTL and GPE - > GEC	0.051	0.050	0.021	2.374	0.018	Accepted
GPE - > GEC	0.334	0.333	0.057	5.819	0.000	Accepted
GTL - > GEC	0.300	0.302	0.058	5.194	0.000	Accepted
GTL - > GHRM	0.162	0.163	0.059	2.755	0.006	Accepted
GTL - > GPE	0.224	0.221	0.059	3.790	0.000	Accepted

### Hypotheses Assessment

Total construct measurement errors and aggregate scores can influence the path coefficients. We used the bias-corrected and accelerated (BCA) confidence interval to control the bias effect (Efron, 1987; Streukens et al., 2010; see Table 8). The bootstrapping method applied 5,000 subsamples at the meaning level of 0.05 to determine standard errors of path coefficient, *p*-values, and t-statistics for the statistical measurement between the hypotheses (Chin, 1998; Hair et al., 2012).

**Table 8:** Indirect effects.

	OM	M	SD	*t*-value	*p*-values	Status
GIS - > GPE - > GEC	0.042	0.045	0.018	2.327	0.020	Accepted
GIS*GTL and GPE - > GPE - > GEC	0.051	0.050	0.021	2.374	0.018	Accepted
GTL - > GHRM - > GEC	0.028	0.029	0.016	1.824	0.069	Accepted
GTL - > GPE - > GEC	0.075	0.073	0.023	3.277	0.001	Accepted

According to the outcomes of Tables 6 and 7, this study revealed a significant direct impact of green transformation leadership on green employee creativity and therefore supported H1 GTL - > GEC (*β* = 0.300; *p* = 0.001). While green transformation leadership also positively influences GHRM and green process engagement, so H2 GTL - > GHRM and H3 GTL - > GPE (*β* = 0.162; *p* = 0.001) and H3 (*β* = 0.224; *p* = 5 0.000) were supported. GHRM and green process engagement had a strong impact on green employee creativity. Therefore, H4 and H5 were supported with H4 (*β* = 0.175; *p* = 5 0.000) H5 (*β* = 0.334; *p* = 5 0.000). Green employee creativity was positively direct influenced by the GHRM and green process engagement. Our findings showed that the GHRM and the green process engagement (as mediator) strongly affected the green transformation leadership and green employee creativity. The results of Tables 6 and 7 demonstrated the importance of GHRM and green processes engagement have a partial mediation between the relationships of green leadership and green employees’ creativity. While in moderation, our results of H6 GIS*GTL and GPE - > GEC (*β* = 0.051; *p* = 0.000) showed that the green innovation strategy strongly influenced the green process engagement. So it means that if the value of green innovation strategy increases, the relationship between green transformation leadership and green process engagement becomes strong. Results of all hypotheses were presented in Table 5 and mediation results in Table 8.

## Discussion

**Figure 2:**
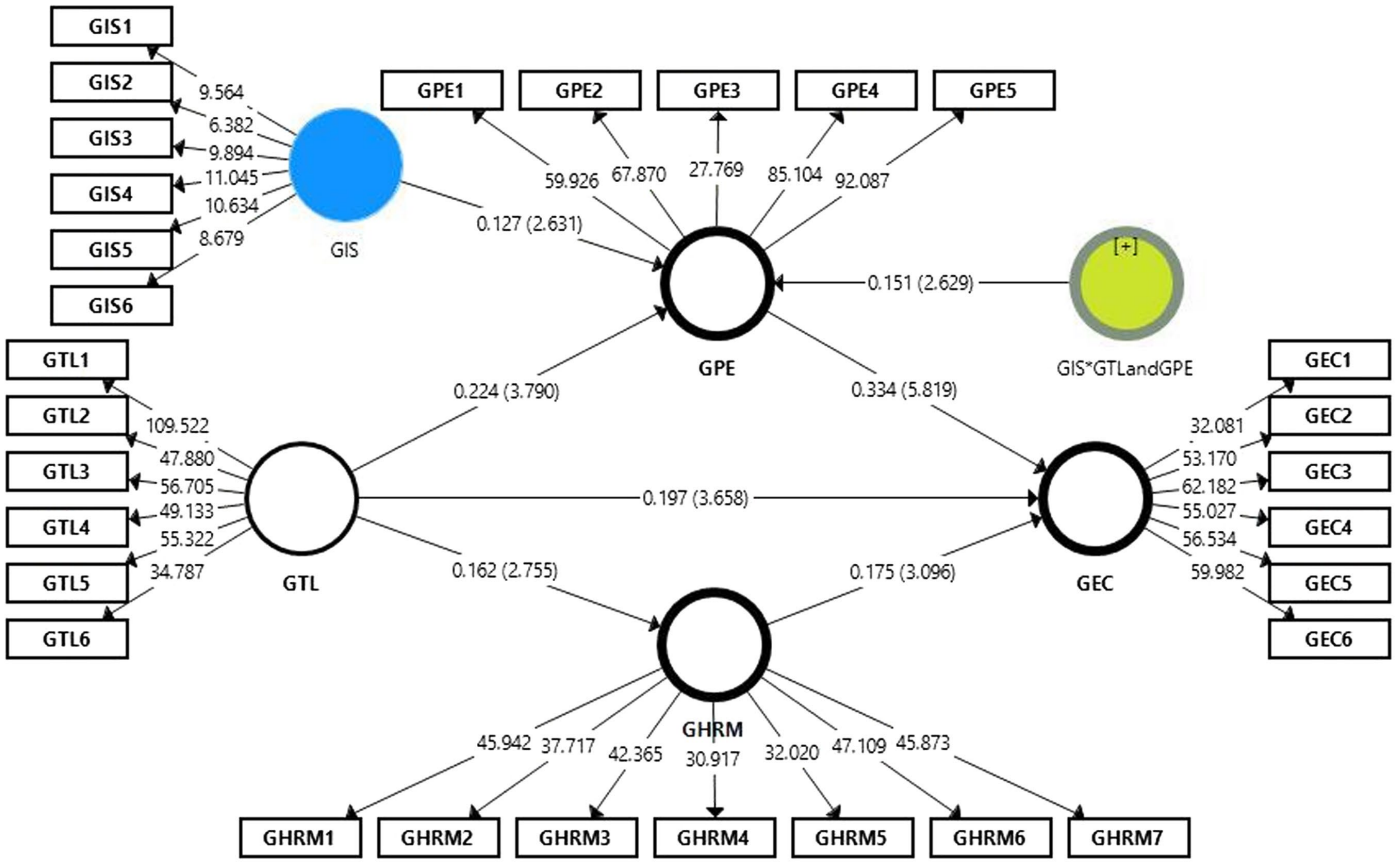
The mediating effect.

The literature is not conclusive on enhancing green employee creativity performance in an integrated framework under environmentalism. All hypothesis relations are accepted based on Tables 6–8 and Figure 2. We offer a green transformational leadership approach to improve green process engagement, GHRM performance, and green employee creativity in the environmental era. Furthermore, we have developed a green transformational leadership research framework to discuss its relationships with green process engagement, green human resource management, and green employee creativity. According to the empirical findings, green transformational leadership is positively related to green creativity, and GHRM increases green employee creativity. We found that green employee creativity mediates positive relationships between green process engagement and GHRM. The results support our hypotheses. Investing in green transformational leadership, green process engagement, and GHRM is beneficial to increasing green employee creativity. Thus, green creativity is critical in determining the performance of green product development. Green transformation leadership would mediate the positive relationships between green process engagement and GHRM, so companies must improve the green creativity of their green product development.

Environmental challenge has become an unavoidable factor. Companies must promote employees’ green creativity to gain a green competitive advantage opportunity improve employees’ ability to enhance the new ecological idea. This study found a positive link between transformational leadership, green creativity, green innovation strategies, green process engagement, and GHRM. In addition, we applied AMO theory to investigate ecological performance and how transformational leadership, green process engagement, GHRM, and green innovation strategy influence employees’ green creativity: overall organizational environments effectiveness and personal attributes. We studied the moderating influence between transformational leadership and green employee creativity by green process engagement and mediating effect between the green transformational leadership and green employee creativity by GHRM. This study expands our understanding of positive features in green transformational leadership by showing the active influences on green creativity. Outcomes showed that green leadership might influence the green creativity of workers and green creativity indirectly affect GHRM and green engagement. Green Innovation strategy also moderated the relation of green transformational leadership and green process engagement. It reinforced this impact when the degree of green innovation strategy is high rather than low.

This research has developed several results that may help build theories for management purposes. The research study supported the idea that workers’ perceptions of their leaders’ transformational strategies to assist them in improving GHRM and their perspectives about their capability to operate creatively have a measurable influence on their creative performance. These findings are supported by Jia et al. (2018). According to research, green transformational leaders encourage green innovation among their subordinates (Gebauer, 2011). Furthermore, it is discovered that green transformational leadership directly relates to green creativity; therefore, managers should be encouraged to improve their leadership style to promote higher green creativity. Previous research has indicated that industrial companies have prioritized creating green transformational leadership to boost green innovation (Hameed et al., 2021).

## Conclusion

This study reinforces the understanding of the consequence of green transformational leadership on green employee creativity by considering the mediating effects of green process engagement and GHRM and the moderated impact of the Green Innovation Strategy mechanism at a higher level. The results provide a template to verify its universality for subsequent research. Green development is widespread worldwide, but companies need to improve employee creativity to promote operational efficiency. Our research offers significant experience in business innovation and green strategic planning by integrating a unique background with a range of green variables. This research investigated the impact of green transformational leadership in fostering green innovation among workers. It has also investigated the function of green human resource management and green innovation strategy in the intervening role. Hence, this research has offered a better knowledge of the causes and consequences of green activities in the business, such as green innovation. The provided results may help direct companies to re-design their policies to create an innovation-driven environment in their company. As a result, company management must advise their workers and restructure their training programs to improve environmental performance and work creatively. This research study makes four practical contributions to the field. First, we demonstrated that improving green transformational leadership could boost green creativity, green process engagement, and GHRM performance. Assume a company wants to improve its green employee creativity performance. In that case, during the strategic planning stage, they should incorporate the concepts of green transformational leadership, GHRM, and green creativity into their long-term environmental strategies. Second, in a more sophisticated product development context, educating experienced leaders of green transformational projects to increase green process engagement and GHRM to increase employee creativity is worthwhile. Third, according to the findings of this study, companies should improve the green creativity of their green product development projects because there is a significant mediation effect between green process engagement and GHRM. Fourth, because green employee creativity is now a practical approach to developing differentiation and positioning strategies, businesses should use it to differentiate and position their products to capture new green markets. To improve their green product company performance, firms must incorporate green transformational leadership, green process engagement, and green creativity into their long-term strategies. Currently, a significantly increased number of senior managers recognize the impact of green improvement on the long-term benefits of businesses. Still, more research is required to continue providing novel clues into strategy implementation.

### Limitations

Three limitations of the study should be highlighted. First of all, our study focused on only one province in Congo, so we should research other provinces/regions and industries (that is, other manufacturing, inter-industries industries, such as IT and tourism). Therefore, we needed to execute in-depth research to generalize future outcomes. Second, while the multilevel analysis can provide certain advantages (Hox et al., 2017), a transversal design still reduces our ability to explain actual cause and effect. More longitudinal studies (like time studies) must therefore be carried out to understand the background of green creativity better. Third, from my point of view, the involvement of people in innovation and the institutional level of the green innovation strategy evaluate the effect of Green Transformation leadership on green creativity (such as green dynamic ability and green self-efficacy). Future research can add additional lenses to promote the sustainability and economic development of the organization.

### Recommendations

This research offers critical recommendations for managers working in organizations. Using green transformational leadership to develop green human resource management would require significant investment to enhance their green creativity. Suppose the company wants to foster green innovation among its workers. In that case, it must integrate the concepts of green transformational leadership and green human resource management into its long-term environmental plans. Green transformational leadership and human resource management may help organizations significantly create environmentally friendly service behavior. They may create a culture that fosters environmentally friendly attitudes by committing more resources, and they can alter the organization’s thinking process toward environmental preservation.

## Data Availability Statement

The raw data supporting the conclusions of this article will be made available by the authors, without undue reservation.

## Ethics Statement

The studies involving human participants were reviewed and approved by Jiangsu University of Science and Technology, Zhenjiang Jiangsu, China. The ethics committee waived the requirement of written informed consent for participation.

## Author Contributions

This idea was given by MS and NW. MS wrote the complete paper. MN analyzed the data. While MF and AS read and approved the final version. All authors contributed to the article and approved the submitted version.

## Funding

This research is supported by the National Science Foundation of China (grant numbers: 71971101 and 71972090) and the Key Project of Philosophy and Social Science Research in Colleges and University of Jiangsu Province (grant number: 2019SJZDA032).

## Conflict of Interest

The authors declare that the research was conducted in the absence of any commercial or financial relationships that could be construed as a potential conflict of interest.

## Publisher’s Note

All claims expressed in this article are solely those of the authors and do not necessarily represent those of their affiliated organizations, or those of the publisher, the editors and the reviewers. Any product that may be evaluated in this article, or claim that may be made by its manufacturer, is not guaranteed or endorsed by the publisher.
